# Influence of interpersonal violence on maternal anxiety, depression, stress and parenting morale in the early postpartum: a community based pregnancy cohort study

**DOI:** 10.1186/1471-2393-12-153

**Published:** 2012-12-15

**Authors:** Lise A Malta, Sheila W McDonald, Kathy M Hegadoren, Carol A Weller, Suzanne C Tough

**Affiliations:** 1 Faculty of Medicine, University of Calgary, Calgary, Alberta, Canada; 2 Department of Paediatrics, University of Calgary, Calgary, Alberta, Canada; 3 Faculty of Nursing, University of Alberta, Edmonton, Alberta, Canada; 4 Department of Community Health Sciences, University of Calgary, Calgary, Alberta, Canada; 5 Child Development Centre, Alberta Children’s Hospital, c/o 2888 Shaganappi Trail, NW, Calgary, Alberta, T3B 6A8, Canada

**Keywords:** Interpersonal violence, Pregnancy cohort, Postpartum mental health, Parenting morale

## Abstract

**Background:**

Research has shown that exposure to interpersonal violence is associated with poorer mental health outcomes. Understanding the impact of interpersonal violence on mental health in the early postpartum period has important implications for parenting, child development, and delivery of health services. The objective of the present study was to determine the impact of interpersonal violence on depression, anxiety, stress, and parenting morale in the early postpartum.

**Methods:**

Women participating in a community-based prospective cohort study (n = 1319) completed questionnaires prior to 25 weeks gestation, between 34–36 weeks gestation, and at 4 months postpartum. Women were asked about current and past abuse at the late pregnancy data collection time point. Postpartum depression, anxiety, stress, and parenting morale were assessed at 4 months postpartum using the Edinburgh Postnatal Depression Scale, the Spielberger State Anxiety Index, the Cohen Perceived Stress Scale, and the Parenting Morale Index, respectively. The relationship between interpersonal violence and postpartum psychosocial health status was examined using Chi-square analysis (p < 0.05) and multivariable logistic regression.

**Results:**

Approximately 30% of women reported one or more experience of interpersonal violence. Sixteen percent of women reported exposure to child maltreatment, 12% reported intimate partner violence, and 12% reported other abuse. Multivariable logistic regression analysis found that a history of child maltreatment had an independent effect on depression in the postpartum, while both child maltreatment and intimate partner violence were associated with low parenting morale. Interpersonal violence did not have an independent effect on anxiety or stress in the postpartum.

**Conclusion:**

The most robust relationships were seen for the influence of child maltreatment on postpartum depression and low parenting morale. By identifying women at risk for depression and low parenting morale, screening and treatment in the prenatal period could have far-reaching effects on postpartum mental health thus benefiting new mothers and their families in the long term.

## Background

Interpersonal violence is a significant risk factor for poor health outcomes in women [1], and is recognized by the World Health Organization as an essential public health priority [2]. Interpersonal violence refers to intentional violence between individuals either in the family or community that is in the form of neglect, psychological or emotional, sexual, or physical violence [2,3]. Family violence often takes place in the home (e.g. child maltreatment or intimate partner violence), while community violence occurs between unrelated individuals (e.g. rape, sexual assault by an unknown person, random acts of violence) and includes violence in institutional settings such as workplaces or schools [2]. Recent Canadian data indicate that over half of Canadian women have survived at least one incident of sexual or physical violence at some point in their lives and 29% have been assaulted by a spouse [4]. True prevalence rates of interpersonal violence is remain elusive, given differences in definitions and types of violence, as well as variations across studies in design, timing of assessment, period versus point prevalence estimates, and under-reporting of incidences of violence [5].

The effects of family violence (child maltreatment or intimate partner violence) on women are extensive andhave long-term consequences [1]. Child maltreatment is a risk for variety of psychological symptoms and psychiatric disorders in adulthood [6-19], as well as long term physical problems [11,14,19-22]. The effects of intimate partner violence includes mental health problems, substance abuse and chronic health problems [23-26]. During pregnancy and the perinatal period, both past and current interpersonal violence may present unique psychological and physical challenges such as fear of childbirth, preterm labour, having a low birth weight infant, neonatal death, and difficulties with breast feeding [27-31].

Given the positive impact of maternal well-being during the postpartum period on healthy parent–child interaction and child development, understanding the factors that influence mental health during the postpartum period is of paramount importance. To date, the majority of studies have focused on specific symptomatology, such as depression, for specific types and timing of abuse. Postpartum depression has been associated with lifetime physical and sexual abuse [32], recent intimate partner violence [33], and traumatic life events [34], although the literature remains inconclusive [35]. Among women with postpartum depression, a history of child maltreatment can exacerbate depressive symptoms which can lead to impaired mother-infant interactions [36], affecting the emotional development of the child [37]. Studies on the effect of interpersonal violence on postpartum anxiety and other emotional problems during the postpartum period are lacking [38]. Maternal anxiety often occurs alongside depression [39,40] but has been less commonly described [41]. However, evidence suggests that maternal anxiety is associated with poorer coping and less confidence in parenting, and affects attachment and bonding [42,43], infant temperament [44,45], and child development [46]. Furthermore, studies to date that have examined interpersonal violence and postpartum psychosocial health have not adequately accounted for potential confounding variables and other risk factors for poor mental health in multivariable analyses. In particular, few studies address maternal physical energy levels when examining postpartum mental health, despite it’s confounding influence, and to our knowledge, none have taken intrinsic traits, like optimism, into consideration.

In summary, there is a need for further research regarding the effect of interpersonal violence on mental health indicators in the postpartum. While some studies have assessed the impact of abuse on postpartum depression, the numbers of studies are limited and results are inconclusive. The aim of this study is to examine the impact of interpersonal violence on women’s mental health in the postpartum period, specifically symptoms of depression, anxiety, stress, and parenting morale, while controlling for potential confounding variables and important risk factors for poor mental health. Study findings may have clinical implications regarding prenatal screening and preventative measures that can be initiated in the prenatal period to support women and improve postpartum mental health.

## Methods

The All Our Babies (AOB) study is a community-based prospective study in Calgary, Alberta that began recruitment in 2008. The overall aims were to examine maternal well-being during the perinatal period and rates of infant outcomes such as preterm birth, and to identify the current barriers and facilitators to accessing prenatal care in Calgary. Women were recruited (n = 1654) from health care offices, community posters, through the Alberta Health Services website, and through Calgary Lab Services. Women were eligible if they were: a) less than 25weeks gestational age at the time of recruitment, b) receiving prenatal care in Calgary, and c) able to complete questionnaires in English. Of those who were eligible (n = 1649), 91% completed at least 1 questionnaire. The most common reasons for discontinuation were pregnancy loss or lost to follow up.

Participation in the study involved the completion of three mailed questionnaires and linkage to prenatal and birth records. The first self-report questionnaire was completed before 25 weeks gestation (mean = 16.4 wks; sd = 4.2wks), the second between 34–36 weeks gestation (mean = 34.5wks; sd = 1.5wks), and the third at 4 months (16wks) postpartum (mean = 17.8wks; sd = 3.3 wks). The questionnaires, which were composed of both standardized scales and investigator derived questions, asked about socio-demographics, lifestyle, heath care utilization, mental health, social support, birth outcomes, postpartum experiences, and breastfeeding, among other variables.

For the purposes of the present study, we restricted the sample to women having complete information across all three data collection time points in the perinatal period (n = 1319). There were no differences in sociodemographic or psychosocial characteristics between the study sample and the larger sample (results not shown). For the present analysis, the following validated instruments were used: the Edinburgh Postnatal Depression Scale (EPDS) [47], the Spielberger State Anxiety Index (SSAI) [48], the Cohen Perceived Stress Scale (PSS) [49], the Parenting Morale Index [50], the Life Orientation Test-Revised [51], and the Medical Outcomes Study Social Support Scale [52] to measure depression, anxiety, stress, parenting morale, optimism, and social support, respectively. To operationalize the postpartum maternal psychological health outcomes, we used established cut-offs as per the literature for symptoms of depression and anxiety (10 or above on the EPDS and 40 or above on the SSAI), and above the 80^th^ percentile or below the 20^th^ percentile or of the distribution for stress and parenting morale.

The main exposure for the present study was interpersonal violence, as assessed at the second data collection time point in late pregnancy (34–36 weeks), according to detailed questions on exposure, timing, and perpetrator to five different types of abuse: physical, sexual, emotional, financial, and neglect. Types of interpersonal violence used in the present study were derived as per national and international classifications and previous research [2,3,53,54], as well as through discussions with content experts in the field: child maltreatment, intimate partner violence, and ‘other’ abuse. Child maltreatment was classified as any abuse that occurred when individuals were younger than 18 years of age. Intimate partner violence was defined as any type of abuse committed by a current or former partner, spouse or ex-spouse, or a current or former dating partner. ‘Other’ abuse included women who noted an experience of abuse but did not provide sufficient details to be classified as either child maltreatment or intimate partner violence, as well as women who experienced abuse outside of an intimate partner relationship as adults (e.g., abuse by work colleagues, friends), and women who experienced sexual assault as adults. Other variables of interest for the present study included sociodemographic and socioeconomic factors, history of mental illness and drug or alcohol abuse, and self-reported energy levels. These variables were assessed mainly using single item questions. See Additional file 1 for a description of select standardized tools as well as phrasing and coding for single item measures.

Descriptive statistics were used to assess the characteristics of study participants. Comparisons between those who reported any type of experience of abuse and those who did not were performed using Chi Square test/Fisher’s exact test for categorical variables. Bivariate analysis was performed to identify potential confounding variables and risk factors for inclusion in multivariable models. Due to multiple testing, an alpha level of 1% was adopted as the threshold for statistical significance. Separate multivariable logistic regression analyses were performed to examine the extent to which exposure to interpersonal violence was an independent predictor of maternal depression, stress, anxiety, and parenting morale at 4 months postpartum, controlling for known risk factors and potential confounding variables. All analyses were performed using SPSS version 19.0 (SPSS Inc. Chicago, Illinois).

This study was approved by the Conjoint Health Research Ethics Board of the University of Calgary. Participants provided informed consent at the time of recruitment and were provided copies for their records.

## Results

Sample characteristics are presented in Table 1. The AOB cohort is well-educated and has above-average affluence, and is comparable to the target population of women who plan to become pregnant and parent in Canada [55,56]. Women who experienced interpersonal violence were more likely to have lower socioeconomic status, single, Caucasian, and Canadian born, compared to women with no reported experiences of interpersonal violence. The proportion of women with a history of mental illness and drug or alcohol abuse was higher among women with exposure to interpersonal violence. Approximately 30% recorded at least one exposure to interpersonal violence. Women with experiences of interpersonal violence reported mental health concerns at 4 months postpartum at rates that were approximately twice as great as those reported by women without experiences of interpersonal violence: depression (22% vs. 10%), anxiety (24% vs. 12%), high perceived stress (24% vs. 14%), and low parenting morale (29% vs. 16%).

**Table 1:** Comparison of demographic and maternal characteristics between women with and without experiences of interpersonal violence (IPV)

**Characteristic**	**Total sample (n = 1382)**	**Experienced IPV (n = 410)**	**No experiences of IPV (n = 972)**
	**n (%)**	**n (%)**	**n (%)**
**Maternal Age**			
<25 yrs	120(8.8)	43(10.7)	77(8.0)
25–34 yrs	990(72.6)	280(69.5)	710(74.0)
35+ yrs	253(18.6)	80(19.9)	173(18.0)
**Education**			
High school or less	139(10.1)	57(14.0)	82(8.5)
Completed degree or some post-Secondary	1232(89.9)	349(86.0)	883(91.5)
**Household Income**			
<60,000	240(18.1)	80(20.1)	160(17.3)
>60,000	1086(81.9)	319(79.9)	767(82.7)
**Marital Status**			
Married/common-law	1291(94.2)	353(87.2)	938(97.2)
Other	79(5.8)	52(12.8)	27(2.8)
**Ethnic Origin**			
White/Caucasian	1041(75.9)	319(78.6)	722(74.8)
Other	330(24.1)	87(24.1)	243(25.2)
**Born in Canada**			
Yes	1048(76.4)	330(81.3)	718(74.3)
No	324(23.6)	76(18.7)	248(25.7)
**Parity**			
0	724(53.1)	221(54.4)	503(52.5)
1+	640(46.9)	185(45.6)	455(47.5)
**History of Mental Health Problems**			
No	898(65.5)	187(46.1)	711(73.6)
Yes	474(34.5)	219(53.9)	255(26.4)
**History of Alcohol or Drug Abuse**			
No	1319(96.1)	366(90.1)	953(98.7)
Yes	53(3.9)	40(9.9)	13(1.3)

Bivariate analysis was completed to determine which sociodemographic and socioeconomic variables, as well as maternal characteristics, were significantly associated with anxiety, depression, stress, and low parenting morale at 4 months postpartum (Table 2). Variables that were significant (p < 0.01) at the bivariate level were considered for inclusion in the multivariable analyses. For each postpartum mental health outcome (e.g. depression, stress, anxiety, and low parenting morale), three separate multivariable regression models were performed – one for each type of interpersonal violence. The reference category for each type of interpersonal violence was no experience of any abuse. All final models controlled for the following covariates and potential confounding variables: household income, history of mental health, maternal optimism, perceived prenatal social support, perceived postpartum social support, and postpartum energy levels. Exposure to child maltreatment was an independent significant risk factor for depression (OR = 1.8, 95% CI = 1.1-3.0) and low parenting morale (OR = 1.8, 95% CI = 1.1-2.8) in the postpartum, controlling for other variables. Exposure to other forms of interpersonal violence did not significantly contribute to the risk for depression or low parenting morale, although the confidence interval for the estimate of intimate partner violence exposure was close to crossing the threshold for significance, with a lower bound of 1.00 (OR = 1.66, 95% CI = 1.00-2.78). Anxiety and stress in the postpartum were not associated with any types of interpersonal violence. Table 3 presents the final multivariable logistic regression models for depression and parenting morale, and each category of interpersonal violence.

**Table 2:** Characteristics of women who experience poor mental health outcomes at 4 months postpartum

**Independent variable**	**Depression (EPDS ≥ 10) n(%)**		**Anxiety (SSAI ≥ 40) n(%)**		**High stress n(%)**		**Low parenting morale n(%)**	
	**Yes (n = 177)**	**No (n = 1170)**	**Yes (n = 206)**	**No (n = 1125)**	**Yes (n = 229)**	**No (n = 1115)**	**Yes (n = 265)**	**No (n = 1074)**
**Maternal Age**								
<25 yrs	19(11.0)*	89(7.7)	21(10.4)	85(7.7)	19(11.0)	80(7.3)	21(8.0)	87(8.2)
25–34 yrs	110(63.6)	863(74.6)	138(68.7)	827(74.4)	110(63.6)	826(75.2)	186(71.0)	779(73.6)
35+ yrs	44(25.4)	203(17.6)	42(20.9)	199(17.9)	44(25.4)	193(17.6)	55(21.0)	192(18.1)
**Education**								
High school or less	24(13.6)	111(9.6)	22(10.7)	114(10.2)	38(16.7)*	100(9.0)	31(11.7)	106(9.9)
Completed degree or some post-Secondary	152(86.4)	1051(90.4)	183(89.3)	1003(89.8)	190(83.3)*	1007(91.0)	233(88.3)	960(90.1)
**Household Income**								
<60,000	51(29.7)*	180(160)	59(29.8)*	170(15.7)	67(30.6)*	165(15.3)	62(24.1)*	170(16.5)
60,000+	121(70.3)*	944(84.0)	139(70.2)*	912(84.3)	152(69.4)*	912(84.7)	195(75.9)*	861(83.5)
**Marital Status**								
Married/common-law	159(90.3)*	1106(95.3)	184(90.2)*	1064(93.5)	208(91.2)	1053(93.3)	246(93.2)	1009(94.8)
Other	17(9.7)*	54(4.7)	20(9.8)*	52(4.7)	20(8.8)	52(4.7)	18(6.8)	55(5.2)
**Ethnic Origin**								
White/Caucasian	125(71.0)	894(77.1)	150(73.5)	860(77.1)	166(72.8)	850(76.9)	210(79.5)	802(75.4)
Other	51(29.0)	266(22.9)	54(26.5)	256(22.9)	62(27.2)	255(23.1)	54(20.5)	262(24.6)
**Born in Canada**								
Yes	128(72.7)	898(77.2)	159(77.6)	861(77.0)	170(74.6)	853(77.0)	205(77.7)	814(76.3)
No	48(27.3)	265(22.8)	26(22.4)	257(23.0)	58(25.4)	255(23.0)	59(22.3)	253(23.7)
**History of Mental Health Problems**	113(64.6)*	337(29.0)*	127(62.3)*	317(28.3)*	129(56.8)*	320(28.9)*	149(56.7)*	299(28.0)*
**History of Alcohol or Drug Abuse**	11(6.3)	32(2.7)	11(5.4)	33(2.9)	13(5.7)	30(2.7)	15(5.7)	29(2.7)
**Low Optimism (below 20**^**th**^**percentile of distribution)**	43(28.7)*	121(12.7)*	57(33.7)*	106(11.5)*	63(33.0)*	102(11.2)*	62(27.1)*	101(11.6)*
**Low Social Support**	75(43.4)*	115(10.0)*	78(39.2)*	110(9.9)*	91(41.0)*	100(9.1)*	96(37.4)*	93(8.8)*
**Low Energy**	75(42.6)*	142(12.1)*	91(44.0)*	126(11.2)*	93(40.8)*	123(11.0)*	118(44.7)*	99(9.2)*

*significant at p < 0.01.

**Table 3:** Final multivariable logistic regression models examining the independent contribution of interpersonal violence to depression and parenting morale at 4 months postpartum

	**Depression, EPDS ≥ 10**			**Low parenting morale**		
	**OR(CI)**			**OR(CI)**		
Income	2.21(1.34-3.65)	1.96(1.15-3.35)	2.07(1.18-3.64)	1.44(0.91-2.30)	1.48(0.91-2.41)	1.60(0.97-2.66)
Past Mental Health Problems	3.10(1.95-4.93)	2.68(1.66-4.34)	2.83(1.71-4.71)	2.11(1.42-3.13)	2.63(1.75-3.96)	2.55(1.65-3.93)
Low Social Support early pregnancy (<25wks)	0.62(0.32-1.20)	0.66(0.33-1.32)	0.81(0.39-1.68)	0.54(0.29-1.00)	0.63(0.34-1.19)	0.55(0.28-1.10)
Low Social Support late pregnancy (34–36 wks)	4.66(2.58-8.44)	4.60(2.47-8.55)	3.94(1.99-7.81)	4.68(2.67-8.18)	3.82(2.15-6.80)	3.83(2.04-7.19)
Optimism	0.94(0.89-0.99)	0.93(0.88-0.98)	0.81(.0.39-1.68)	0.90(0.86-0.94)	0.90(0.86-0.94)	0.90(0.85-0.94)
Low Energy	2.80(1.71-4.59)	3.37(2.04-5.56)	2.61(1.49-4.55)	4.92(3.18-7.62)	4.75(3.04-7.41)	4.01(2.49-6.47)
Exposure to Child Maltreatment	1.83(1.11-2.99)*			1.78(1.13-2.81)*		
Exposure to Partner violence		1.66(0.95-2.90)			1.66(1.00-2.78)	
Exposure to Other Abuse			1.93(0.81-4.64)			0.92(0.38-2.24)

*significant at p < 0.05.

## Discussion

This study among relatively affluent and well-educated Canadian women found that 29.7% of women had experienced at least one incident of interpersonal violence at some point in their lives. A recent Canadian study of postpartum women showed that 30% reported adult emotional abuse, 7% reported adult physical abuse, 13% reported adult sexual abuse, and 14% and 7% reported child sexual and physical abuse, respectively [57]. Although it is difficult to compare prevalence rates across populations, the prevalence of interpersonal violence in this study and other studies reveals that exposure to interpersonal violence cannot be predicted by adverse socioeconomic circumstances or maternal education. Results from multivariable regression analyses showed that that a history of child maltreatment had an independent effect on depression in the postpartum, while both child maltreatment and intimate partner violence were associated with low parenting morale. Interpersonal violence did not have an independent effect on anxiety or stress in the postpartum.

Exposure to child maltreatment was an independent risk factor for postpartum depression, even while controlling for other factors that are known to influence postpartum depression such as a history of mental illness, social support, and income [58]. There is strong evidence in the literature that child maltreatment is associated with adult depression [9-15], but few studies have examined the association between child maltreatment and postpartum depression. To our knowledge, no studies have considered *all* types of child maltreatment in a general population of postpartum women. In one of the few studies that have been done on this topic, more severe depression was noted among women with a history of child abuse [36]. Two studies have found an association between child sexual and physical abuse and postpartum depression [32] and depressive symptoms [34]. However, both of these studies had small sample sizes, and did not consider other forms of child maltreatment such as psychological abuse and neglect.

Both child maltreatment and intimate partner violence were significantly associated with low parenting morale at 4 months postpartum. Parenting morale is an outcome that has not been considered previously in postpartum women in the literature, but can be useful in understanding parenting enthusiasm or attitudes of women to the parenting role [59]. Previous research has shown that abused women had a more negative parent–child interaction observed in an assessment of parenting stress [36], and research has shown that abusive experiences have a range of negative effects on parenting such as poorer maternal-child interaction, more psychological aggression and physical punishment, less parental warmth, a negative view of self as a parent, decreased parenting satisfaction, problems establishing boundaries as parents, and being too permissive as parents [60-68]. Research indicates that a mother’s current state of mind regarding the abuse and whether the abuse has been resolved is important in experiencing a more healthy adjustment to parenthood [69]. In light of these findings, and the results of this study, it may be useful to identify women with histories of child maltreatment and provide support during the antenatal period to help these women navigate a healthy transition as parents in the postpartum period.

Major strengths of this study refer to its inclusion of all possible types of abuse throughout a woman’s life and its examination of important mental health outcomes that are relatively understudied in postpartum women. For example, this study included psychological abuse, which is often ignored in research and policies, but may have an even greater negative impact on women than physical abuse [70]. Other strengths of the study include its community-based sample of women (rather than a clinical sample), relatively large sample size, and prospective nature of data collection, with interpersonal violence information obtained during pregnancy before the assessment of postpartum depression and parenting morale. In multivariable analyses, this study was able to adjust for a number of relevant confounding variables across the perinatal period, such as fatigue, optimism, and social support, which allowed for a more detailed understanding of the independent influence of interpersonal violence.

Study limitations include the use of several models that were run simultaneously, which could have resulted in an increased risk for type 1 error. However, given the number and quality of adjustment variables used in the multivariable models, independent effects of the interpersonal violence exposure variables for depression and parenting morale suggest that they are robust. Other limitations include the tendency of underreporting that occurs among victims of abuse, as well as barriers that prevent women from disclosing details of their experiences of interpersonal violence. Examples of possible barriers are a hesitancy to discuss traumatic experiences and a fear of disclosing information regarding abuse. Indeed, 7% of women in the study reported at least one incident of abuse, yet did not provide sufficient details for the abusive experiences to be classified as child maltreatment or intimate partner violence. In an effort to not exclude these women from the study or discount their traumatic experiences, they were categorized as ‘other abuse’, along with women who experienced other types of abusive experiences as adults or sexual assault as adults (1.7%). For the purpose of this analysis, dating violence was included in the category of intimate partner violence, which has been supported by the Centers for Disease Control [53], but is differentiated by WHO [2,3]. Although we cannot discount exposure misclassification, the collection of detailed information on experiences of interpersonal violence in the present study allowed for the differentiation and examination of different types of experiences, albeit crude, not seen in previous studies. This information was especially useful for classify past abuse experiences. Unfortunately the level of detail was not enough to fully understand more recent and/or current abuse experiences. For example, we could not distinguish whether interpersonal violence resulted in any of the pregnancies in our study (i.e., sexual assault), nor were we able to fully differentiate current and past intimate partner violence. Despite this, our ability to examine both child maltreatment and intimate partner violence in the same study in relation to a range of postpartum mental health outcomes adds to the evidence base in this area.

There are limitations associated with the use of the SSAI and the EPDS. The SSAI assessed state anxiety only, not general symptoms of anxiety. Although not employed in this study, the trait subscale of the Spielberger Anxiety Inventory may have provided additional information to help in the assessment of anxiety. The cut-off scores for the EPDS in the literature are 10 and 13. In this study, a score of 10 was used to identify women who are experiencing distress and who may be at risk of major depression. Although there is a greater likelihood of including false-positives when using a cut-off score of 10 instead of 13, use of the latter may have led to the exclusion of women with milder symptomatology, whose distress or depression needs identification and support, and whose parenting or ability to bond with their newborn may be compromised by depressive mood.

The results of this study provide direction for further research in this area, including validation of study findings. Further quantitative studies as well as qualitative studies are needed to understand the full impact of previous experiences of violence on mothering and how to provide specific services to this population of women during pregnancy and the postpartum period. Study findings have implications for pregnancy and postpartum care as they indicate that a history of child maltreatment may be an important consideration in prenatal screening as well as prenatal and postpartum services. While it has been recommended that adult women in the general population be questioned about past abusive experiences, including child abuse [71,72], a history of child maltreatment is not routinely considered during prenatal or postpartum care since assessments by clinicians focus on current domestic violence. The Society of Obstetrics and Gynaecology Canada clinical practice guidelines recommend that health care providers ask about intimate partner violence during assessment of new patients, as a part of prenatal care, at annual preventative visits, and if symptoms or conditions are present which are linked with interpersonal violence. However, evidence suggests that only 22.4% and 25.7% of women are asked about emotional and physical abuse, respectively, during pregnancy [73]. Consideration of child maltreatment in screening and follow-up could help to identify women who are at risk, and additional treatment and support may reduce the burden of depression and parenting difficulties in the postpartum period for these women. An emerging area of research in antenatal screening for postpartum depression refers to psychosocial assessment tools that incorporate questions across a number of domains of functioning and experiences in a woman’s life beyond current symptomatology. An important area of focus in these tools includes a woman’s past history of adverse child experiences and abuse. A number of recent studies in this area advocate for routine psychosocial assessment as part of a revised perinatal mental health agenda [74,75]. The results of this study suggest that experiences of interpersonal violence constitute an important area to consider as part of a comprehensive examination of a woman’s risk profile.

## Conclusion

In conclusion, child maltreatment is an important risk factor for postpartum depression and low parenting morale that needs to be identified and assessed by clinicians. Identifying women in the prenatal period who are at risk of depression and low parenting morale may allow for targeted strategies to support women and optimize child development. Further research is needed to validate the important impact that child maltreatment appears to have on mothers in the postpartum period, and investigate possible changes in prenatal care to better meet the needs of women who have experiences of interpersonal violence.

## Competing interests

The authors declare that they have no competing interests.

## Authors’ contributions

LM performed the literature review and drafted the manuscript; SM assisted in statistical analysis and interpretation of results; KM participated in interpretation of results and contributed content expertise; CW assisted in interpretation of results and coding of interpersonal violence; ST conceived the design of the study and participated in all phases of the manuscript. All authors read and approved the final manuscript.

## Pre-publication history

The pre-publication history for this paper can be accessed here:

http://www.biomedcentral.com/1471-2393/12/153/prepub

## Supplementary Material

Additional file 1 Select variables assessed in the All Our Babies study and timing of data collection. Click here for file

## References

[B1] Stewart DE The international consensus statement on women's mental health and the WPA consensus statement on interpersonal violence against women World Psychiatry 2006 5 61 64 16855680 PMC1472251

[B2] World Health Organization World Report on Violence and Health 2002 Geneva, Switzerland: World Health Organization

[B3] World Health Organization Preventing Child Maltreatment: A guide to taking action and generating evidence 2006 Geneva, Switzerland: World Health Organization

[B4] Statistics Canada The Violence Against Women Survey 1993 Ottawa, ON: Statistics Canada 12295372

[B5] Swahn MH Whitaker DJ Pippen CB Leeb RT Teplin LA Abram KM McClelland GM Concordance between self-reported maltreatment and court records of abuse or neglect among high-risk youths Am J Public Health 2006 96 1849 1853 10.2105/AJPH.2004.058230 17008582 PMC1586157

[B6] Briere J Elliott DM Prevalence and psychological sequelae of self-reported childhood physical and sexual abuse in a general population sample of men and women Child Abuse Negl 2003 27 1205 1222 10.1016/j.chiabu.2003.09.008 14602100

[B7] Gao W Paterson J Abbott M Carter S Iusitini L Donald-Sundborn G Impact of current and past intimate partner violence on maternal mental health and behaviour at 2 years after childbirth: evidence from the Pacific Islands Families Study Aust N Z J Psychiatry 2010 44 174 182 10.3109/00048670903487126 20113306

[B8] Greenfield EA Marks NF Identifying experiences of physical and psychological violence in childhood that jeopardize mental health in adulthood Child Abuse Negl 2010 34 161 171 10.1016/j.chiabu.2009.08.012 20223518 PMC2838932

[B9] Cheasty M Clare AW Collins C Relation between sexual abuse in childhood and adult depression: case–control study BMJ 1998 316 198 201 10.1136/bmj.316.7126.198 9468687 PMC2665415

[B10] Kendler KS Bulik CM Silberg J Hettema JM Myers J Prescott CA Childhood sexual abuse and adult psychiatric and substance use disorders in women: an epidemiological and cotwin control analysis Arch Gen Psychiatry 2000 57 953 959 10.1001/archpsyc.57.10.953 11015813

[B11] Springer KW Sheridan J Kuo D Carnes M Long-term physical and mental health consequences of childhood physical abuse: results from a large population-based sample of men and women Child Abuse Negl 2007 31 517 530 10.1016/j.chiabu.2007.01.003 17532465 PMC3031095

[B12] Molnar BE Berkman LF Buka SL Psychopathology, childhood sexual abuse and other childhood adversities: relative links to subsequent suicidal behaviour in the US Psychol Med 2001 31 965 977 11513382 10.1017/s0033291701004329

[B13] Chapman DP Whitfield CL Felitti VJ Dube SR Edwards VJ Anda RF Adverse childhood experiences and the risk of depressive disorders in adulthood J Affect Disord 2004 82 217 225 10.1016/j.jad.2003.12.013 15488250

[B14] Kendall-Tackett KA Physiological correlates of childhood abuse: chronic hyperarousal in PTSD, depression, and irritable bowel syndrome Child Abuse Negl 2000 24 799 810 10.1016/S0145-2134(00)00136-8 10888019

[B15] Sesar K Simic N Barisic M Multi-type childhood abuse, strategies of coping, and psychological adaptations in young adults Croat Med J 2010 51 406 416 10.3325/cmj.2010.51.406 20960590 PMC2969135

[B16] Appleyard K Berlin LJ Rosanbalm KD Dodge KA Preventing early child maltreatment: implications from a longitudinal study of maternal abuse history, substance use problems, and offspring victimization Prev Sci 2011 12 139 149 10.1007/s11121-010-0193-2 21240556 PMC3707116

[B17] Schneider R Baumrind N Kimerling R Exposure to child abuse and risk for mental health problems in women Violence Vict 2007 22 620 631 10.1891/088667007782312140 18064973

[B18] Allison KC Grilo CM Masheb RM Stunkard AJ High self-reported rates of neglect and emotional abuse, by persons with binge eating disorder and night eating syndrome Behav Res Ther 2007 45 2874 2883 10.1016/j.brat.2007.05.007 17659255 PMC2134835

[B19] Springer KW Sheridan J Kuo D Carnes M The long-term health outcomes of childhood abuse. An overview and a call to action J Gen Intern Med 2003 18 864 870 10.1046/j.1525-1497.2003.20918.x 14521650 PMC1494926

[B20] Gilbert R Widom CS Browne K Fergusson D Webb E Janson S Burden and consequences of child maltreatment in high-income countries Lancet 2009 373 68 81 10.1016/S0140-6736(08)61706-7 19056114

[B21] Taylor RR Jason LA Chronic fatigue, abuse-related traumatization, and psychiatric disorders in a community-based sample Soc Sci Med 2002 55 247 256 10.1016/S0277-9536(01)00168-X 12144139

[B22] Walsh CA Jamieson E Macmillan H Boyle M Child abuse and chronic pain in a community survey of women J Interpers Violence 2007 22 1536 1554 10.1177/0886260507306484 17993640

[B23] Dutton MA Green BL Kaltman SI Roesch DM Zeffiro TA Krause ED Intimate partner violence, PTSD, and adverse health outcomes J Interpers Violence 2006 21 955 968 10.1177/0886260506289178 16731994

[B24] Ellsberg M Jansen HA Heise L Watts CH Garcia-Moreno C Intimate partner violence and women's physical and mental health in the WHO multi-country study on women's health and domestic violence: an observational study Lancet 2008 371 1165 1172 10.1016/S0140-6736(08)60522-X 18395577

[B25] Macy RJ Ferron J Crosby C Partner violence and survivors' chronic health problems: informing social work practice Soc Work 2009 54 29 43 10.1093/sw/54.1.29 19205255

[B26] Gottlieb AS Intimate partner violence: a clinical review of screening and intervention Womens Health 2008 4 529 539 10.2217/17455057.4.5.529 19072491

[B27] Lukasse M Vangen S Oian P Kumle M Ryding EL Schei B Childhood abuse and fear of childbirth-a population-based study Birth 2010 37 267 274 10.1111/j.1523-536X.2010.00420.x 21083717

[B28] Curry MA Perrin N Wall E Effects of abuse on maternal complications and birth weight in adult and adolescent women Obstet Gynecol 1998 92 530 534 10.1016/S0029-7844(98)00258-0 9764624

[B29] Sarkar NN The impact of intimate partner violence on women's reproductive health and pregnancy outcome J Obstet Gynaecol 2008 28 266 271 10.1080/01443610802042415 18569465

[B30] Shumway J O'Campo P Gielen A Witter FR Khouzami AN Blakemore KJ Preterm labor, placental abruption, and premature rupture of membranes in relation to maternal violence or verbal abuse J Matern Fetal Med 1999 8 76 80 10338059 10.1002/(SICI)1520-6661(199905/06)8:3<76::AID-MFM2>3.0.CO;2-C

[B31] Roussillon JA Adult survivors of childhood sexual abuse: suggestions for perinatal caregivers Clin Excell Nurse Pract 1998 2 329 337 12596835

[B32] Records K Rice MJ Lifetime physical and sexual abuse and the risk for depression symptoms in the first 8 months after birth J Psychosom Obstet Gynaecol 2009 30 181 190 10.1080/01674820903178121 19728219

[B33] Beydoun HA Al-Sahab B Beydoun MA Tamim H Intimate partner violence as a risk factor for postpartum depression among Canadian women in the maternity experience survey Ann Epidemiol 2010 20 575 583 10.1016/j.annepidem.2010.05.011 20609336 PMC4179881

[B34] Mezey G Bacchus L Bewley S White S Domestic violence, lifetime trauma and psychological health of childbearing women BJOG 2005 112 197 204 10.1111/j.1471-0528.2004.00307.x 15663584

[B35] Records K Rice MJ A comparative study of postpartum depression in abused and non-abused women Arch Psychiatr Nurs 2005 19 281 290 10.1016/j.apnu.2005.07.010 16308128

[B36] Buist A Childhood abuse, parenting and postpartum depression Aust N Z J Psychiatry 1998 32 479 487 10.3109/00048679809068320 9711360

[B37] Buist A Janson H Childhood sexual abuse, parenting and postpartum depression-a 3-year follow-up study Child Abuse Negl 2001 25 909 921 10.1016/S0145-2134(01)00246-0 11523868

[B38] Barber CC Perinatal mental health care in New Zealand: the promise of beginnings New Zeal J Psychol 2009 38 32 38

[B39] Austin MP Hadzi-Pavlovic D Priest SR Reilly N Wilhelm K Saint K Parker G Depressive and anxiety disorders in the postpartum period: how prevalent are they and can we improve their detection? Arch Womens Ment Health 2010 13 395 401 10.1007/s00737-010-0153-7 20232218

[B40] Matthey S Barnett B Howie P Kavanagh DJ Diagnosing postpartum depression in mothers and fathers: whatever happened to anxiety? J Affect Disord 2003 74 139 147 10.1016/S0165-0327(02)00012-5 12706515

[B41] Yelland J Sutherland G Brown SJ Postpartum anxiety, depression and social health: findings from a population-based survey of Australian women BMC Public Health 2010 10 771 10.1186/1471-2458-10-771 21167078 PMC3022850

[B42] Feldman R Weller A Leckman JF Kuint J Eidelman AI The nature of the mother's tie to her infant: maternal bonding under conditions of proximity, separation, and potential loss J Child Psychol Psychiatry 1999 40 929 939 10.1017/S0021963099004308 10509887

[B43] Nagata M Nagai Y Sobajima H Ando T Nishide Y Honjo S Maternity blues and attachment to children in mothers of full-term normal infants Acta Psychiatr Scand 2000 101 209 217 10.1046/j.0902-4441.2000.ap90075.x 10721869

[B44] McMahon C Barnett B Kowalenko N Tennant C Don N McMahon C Barnett B Kowalenko N Tennant C Don N Postnatal depression, anxiety and unsettled infant behaviour Aust N Z J Psychiatry 2001 35 581 588 200 10.1080/0004867010060505 11551272

[B45] Burke JG Lee LC O'Campo P An exploration of maternal intimate partner violence experiences and infant general health and temperament Matern Child Health J 2008 12 172 179 10.1007/s10995-007-0218-z 17549615

[B46] Galler JR Harrison RH Ramsey F Forde V Butler SC Maternal depressive symptoms affect infant cognitive development in Barbados J Child Psychol Psychiatry 2000 41 747 757 10.1111/1469-7610.00662 11039687

[B47] Cox J Holden J Sagovsky R Detection of postnatal depression. Development of the 10-item Edinburgh Postnatal Depression Scale Br J Psychiatry 1987 150 782 786 10.1192/bjp.150.6.782 3651732

[B48] Spielberger C Gorsuch R Test Manual for the State-Trait Anxiety Inventory 1970 Palo Alto, California: Consulting Psychologist's Press

[B49] Cohen S Kamarck T Mermelstein R A global measure of perceived stress J Health Soc Behav 1983 24 385 396 10.2307/2136404 6668417

[B50] Trute B Hiebert-Murphy D Predicting family adjustment and parenting stress in childhood disability services using brief assessment tools J Intellect Dev Disabil 2005 30 217 225 10.1080/13668250500349441

[B51] Scheier MF Carver CS Bridges MW Distinguishing optimism from neuroticism (and trait anxiety, self-mastery, and self-esteem): a reevaluation of the Life Orientation Test J Pers Soc Psychol 1994 67 1063 1078 7815302 10.1037//0022-3514.67.6.1063

[B52] Sherbourne CD Stewart AL The MOS social support survey Soc Sci Med 1991 32 705 714 10.1016/0277-9536(91)90150-B 2035047

[B53] Saltzman L Fanslow J McMahon P Shelley G Intimate Partner Violence Surveillance: Uniform Definitions and Recommended Data Elements 2002 Atlanta, Georgia: Centers for Disease Control and Prevention, National Center for Injury Prevention and Control

[B54] Beydoun HA Tamim H Lincoln AM Dooley SD Beydoun MA Association of physical violence by an intimate partner around the time of pregnancy with inadequate gestational weight gain Soc Sci Med 2011 72 867 873 10.1016/j.socscimed.2011.01.006 21324411 PMC3443557

[B55] Chalmers B Dzakpasu S Kaczorowski J The maternity experiences survey: an overview of findings J Obstet Gynecol Can 2008 30 217 228 18364099 10.1016/S1701-2163(16)32758-X

[B56] Gracie SK Lyon AW Kehler HL Pennell CE Dolan SM McNeil DA Siever JE McDonald SW Bocking AD Lye SJ Hegadoren KM Olson DM Tough SC All Our Babies Cohort Study: recruitment of a cohort to predict women at risk of preterm birth through the examination of gene expression profiles and the environment BMC Pregnancy Childbirth 2010 10 87 10.1186/1471-2393-10-87 21192811 PMC3022739

[B57] Ansara D Cohen MM Gallop R Kung R Schei B Predictors of women's physical health problems after childbirth J Psychosom Obstet Gynaecol 2005 26 115 125 10.1080/01443610400023064 16050537

[B58] Robertson E Grace S Wallington T Stewart DE Antenatal risk factors for postpartum depression: a synthesis of recent literature Gen Hosp Psychiatry 2004 26 289 295 10.1016/j.genhosppsych.2004.02.006 15234824

[B59] Pitre NY Kushner KE Hegadoren KM The search for safety, control, and voice for mothers living with the legacy of childhood violence experiences. A critical feminist narrative inquiry Adv Nurs Sci 2011 34 260 275 10.1097/ANS.0b013e31822723ed 21822073

[B60] Fitzgerald MM Shipman KL Jackson JL McMahon RJ Hanley HM Perceptions of parenting versus parent–child interactions among incest survivors Child Abuse Negl 2005 29 661 681 10.1016/j.chiabu.2004.10.012 15979708

[B61] Cole PM Woolger C Power TG Smith KD Parenting difficulties among adult survivors of father-daughter incest Child Abuse Negl 1992 16 239 249 10.1016/0145-2134(92)90031-L 1559172

[B62] Barrett B The impact of childhood sexual abuse and other forms of childhood adversity on adulthood parenting J Child Sex Abus 2009 18 489 512 10.1080/10538710903182628 20183414

[B63] Letourneau N Young C Secco L Stewart M Hughes J Critchley K Supporting mothering: service providers' perspectives of mothers and young children affected by intimate partner violence Res Nurs Health 2011 34 192 203 10.1002/nur.20428 21391219

[B64] Peled E Gil IB The mothering perceptions of women abused by their partner Violence Against Women 2011 17 457 479 10.1177/1077801211404676 21478221

[B65] Banyard VL The impact of childhood sexual abuse and family functioning on four dimensions of women's later parenting Child Abuse Negl 1997 21 1095 1107 10.1016/S0145-2134(97)00068-9 9422829

[B66] Banyard VL Williams LM Siegel JA The impact of complex trauma and depression on parenting: an exploration of mediating risk and protective factors Child Maltreat 2003 8 334 349 10.1177/1077559503257106 14604179

[B67] DiLillo D Damashek A Parenting characteristics of women reporting a history of childhood sexual abuse Child Maltreat 2003 8 319 333 10.1177/1077559503257104 14604178

[B68] Lang AJ Gartstein MA Rodgers CS Lebeck MM The impact of maternal childhood abuse on parenting and infant temperament J Child Adolesc Psychiatr Nurs 2010 23 100 110 10.1111/j.1744-6171.2010.00229.x 20500626

[B69] Leon K Jacobvitz D Hazen N Maternal resolution of loss and abuse: associations with adjustment to the transition to parenthood INF MENTAL HLTH J 2004 25 130 148

[B70] O'Leary KD Psychological abuse: a variable deserving critical attention in domestic violence Violence Vict 1999 14 3 23 10397623

[B71] Coid J Petruckevitch A Chung WS Richardson J Moorey S Feder G Abusive experiences and psychiatric morbidity in women primary care attenders Br J Psychiatry 2003 183 332 339 10.1192/bjp.183.4.332 14519611

[B72] Lutenbacher M Relationships between psychosocial factors and abusive parenting attitudes in low-income single mothers Nurs Res 2002 51 158 167 10.1097/00006199-200205000-00004 12063414

[B73] Cherniak D Grant L Mason R Moore B Pellizari R Intimate partner violence consensus statement J Obstet Gynecol Can 2005 27 365 418 15999433 10.1016/s1701-2163(16)30465-0

[B74] Priest SR Austin MP Barnett BB Buist A A psychosocial risk assessment model (PRAM) for use with pregnant and postpartum women in primary care settings Arch Womens Ment Health 2008 11 307 317 10.1007/s00737-008-0028-3 18726142

[B75] Matthey S Phillips J White T Glossop P Hopper U Panasetis P Petridis A Larkin M Barnett B Routine psychosocial assessment of women in the antenatal period: frequency of risk factors and implications for clinical services Arch Womens Ment Health 2004 7 223 229 10.1007/s00737-004-0064-6 15338316

