# Supplementary material for: Influence of interpersonal violence on maternal anxiety, depression, stress and parenting morale in the early postpartum: a community based pregnancy cohort study
Source: BMC Pregnancy Childbirth. 2012 Dec 15;12:153. doi: 10.1186/1471-2393-12-153 (PMC3544728; doi:10.1186/1471-2393-12-153)
Supplement: Additional file 1 — Select variables assessed in the All Our Babies study and timing of data collection. [file 1471-2393-12-153-S1.docx]

Select variables assessed in the All Our Babies study and timing of data collection

| Candidate variable | Source/phrasing | Scoring and/or coding information |
| --- | --- | --- |
| Standardized Scales |  |  |
| Depression (4 months postpartum) | Edinburgh Postnatal Depression Scale | 10 item questionnaire. Each item rated on a 4-point Likert scale from 0-3. After reverse scoring for some items, a total score is derived (range 0-30). Higher scores reflect increased depression. Standard cut-offs include 10 (general distress and at risk for major depression) or 13 (major depression) as per the literature. A cut-off of 10 was used in the present study. |
| Anxiety (4 months postpartum) | State-Trait Anxiety Inventory (state anxiety scale) | 20 item questionnaire. Each item rated on a 4-point Likert scale from 1-4. After reverse scoring for some items, a total score is derived (range 20-80). Higher scores reflect increased anxiety. Anestablished cut-off of 40 was used to classify women as anxious. |
| Perceived Stress (4 months postpartum) | Perceived Stress Scale | 10 item questionnaire. Each item rated on a 5-point Likert scale from 0-4. After reverse scoring for some items, a total score is derived (range 0-40). Higher scores reflect increased stress. A cut-off at the 80^th^ percentile of the sample distribution was used to classify women as stressed. |
| Parenting Morale (4 months postpartum) | Parenting Morale Index | 10 item questionnaire. Each item rated on a 5-point Likert scale from 1-5.After reverse scoring for some items, a total score is derived (range 10-50). Lower scores reflect lower parenting morale. A cut-off at the 20^th^ percentile of the sample distribution was used to classify women as having low parenting morale. |
| Optimism in late pregnancy (34-36 weeks) | Life Orientation Test-Revised | 10 item scale, with 4 items used as fillers and not included in the final score. Each item is scored between 0 and 4. After reverse scoring for some items, a total score is derived (0-24). Higher scores represent greater levels of optimism. |
| Single-item questions |  |  |
| History of alcohol or drug abuse (<25 weeks) | Two single item questions: “Have you ever had alcohol dependency problems?” and “Have you ever had drug dependency problems (including prescription medications)? | Yes/No |
| History of mental health problems (<25 weeks) | Two single item questions: “Have you ever experienced feeling sad, blue, depressed or down for most of the time for at least 2 weeks?” and “Have you ever experienced other mental disorders such as generalized anxiety disorder, bipolar disorder, schizophrenia, or obsessive compulsive disorder?” | Yes/No |
| History of interpersonal violence (34-36 weeks) | Five questions (with subquestions) developed for this study that assessed a history of physical, emotional, sexual, and financial abuse, and neglect. | Yes/No to five questions on history of different types of abuse. A final ‘presence of abuse’ variable was derived by summing across the five types. |
| Low Energy (4 months postpartum) | Single item question: “During the past 4 weeks, how much of the time did you have a lot of energy?” | 5 response choices: All of the time, Most of the time, Some of the time, A little of the time, None of the time. Final coding combined choices ‘A little of the time’ and ‘None of the time’ as low energy. |
